# Thermal conductivity of hexagonal BC_2_P – a first-principles study†

**DOI:** 10.1039/d0ra08444a

**Published:** 2020-11-23

**Authors:** Rajmohan Muthaiah, Fatema Tarannum, Roshan Sameer Annam, Avinash Singh Nayal, Swapneel Danayat, Jivtesh Garg

**Affiliations:** School of Aerospace and Mechanical Engineering, University of Oklahoma Norman OK-73019 USA rajumenr@ou.edu

## Abstract

In this work, we report a high thermal conductivity (*k*) of 162 W m^−1^ K^−1^ and 52 W m^−1^ K^−1^ at room temperature, along the directions perpendicular and parallel to the *c*-axis, respectively, of bulk hexagonal BC_2_P (h-BC_2_P), using first-principles calculations. We systematically investigate elastic constants, phonon group velocities, phonon linewidths and mode thermal conductivity contributions of transverse acoustic (TA), longitudinal acoustic (LA) and optical phonons. Interestingly, optical phonons are found to make a large contribution of 30.1% to the overall *k* along a direction perpendicular to the *c*-axis at 300 K. BC_2_P is also found to exhibit high thermal conductivity at nanometer length scales. At 300 K, a high *k* value of ∼47 W m^−1^ K^−1^ is computed for h-BC_2_P at a nanometer length scale of 50 nm, providing avenues for achieving efficient nanoscale heat transfer.

## Abstract 2

In this work, we report a high thermal conductivity (*k*) of 162 W m^−1^ K^−1^ and 52 W m^−1^ K^−1^ at room temperature, along the directions perpendicular and parallel to the *c*-axis, respectively, of bulk hexagonal BC_2_P (h-BC_2_P), using first-principles calculations.

## Graphical abstract

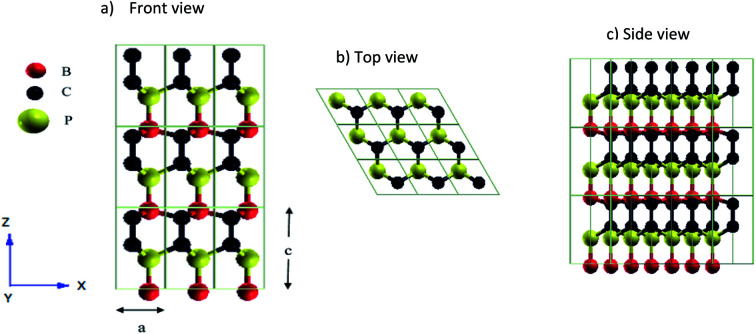

## Introduction

High thermal conductivity materials are crucial for achieving efficient thermal management in electronics to improve both performance and reliability.^1–8^ Carbon based materials such as diamond,^9–11^ graphene^12–14^ and stacked-graphene^15^ (graphene nanoplatelets) exhibit ultrahigh thermal conductivity due to the light mass of carbon (C) atoms and strong C–C bonds. Likewise, boron based III–V compound semiconductors such as boron nitride (BN),^16^ boron phosphide (BP)^17–19^ and boron arsenide (BAs)^20,21^ have very high thermal conductivity due to the light mass of boron atoms and due to a phonon bandgap in vibrational spectra of these materials which suppresses scattering of acoustic phonons by optical phonons thus leading to high acoustic phonon lifetimes. Recently, ultra-high thermal conductivities of 2305 W m^−1^ K^−1^ and 4196 W m^−1^ K^−1^ were reported^22^ for bulk ultra-hard hexagonal BC_2_N (h-BC_2_N) at 0 GPa and 150 GPa respectively. Similarly, for monolayer BC_2_N, high thermal conductivities of 1275.79 W m^−1^ K^−1^ and 893.9 W m^−1^ K^−1^ were reported along the zigzag and armchair directions, respectively.^23^ These results provide motivation to further explore thermal conductivity of III–IV–V compounds. In this work we explore thermal conductivity of hexagonal BC_2_P.

**Fig. 1:**
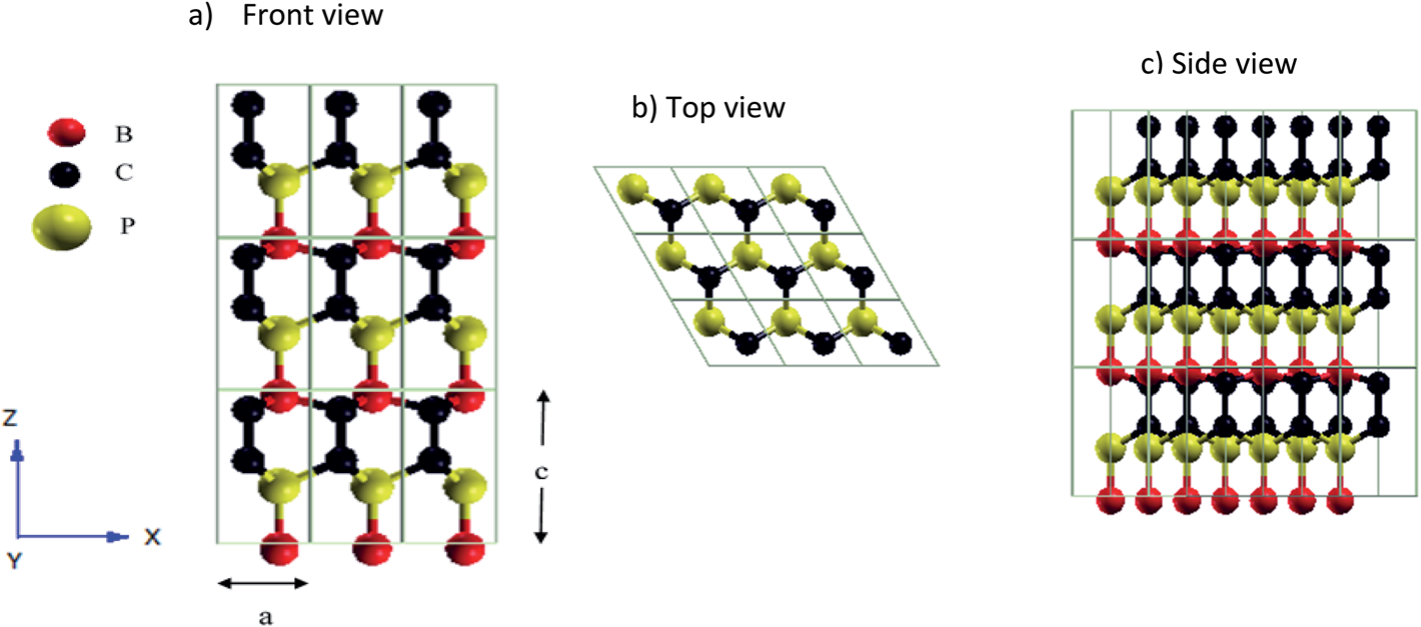
Atomic arrangements of h-BC_2_P with 3 × 3 × 3 supercell with lattice constants *a* = 5.2686 bohr and *c*/*a* = 1.686 (a) front view (b) top view and (c) side view. Red, black and yellow spheres represent boron, carbon and phosphorous atoms respectively.

In this work, thermal conductivity of bulk hexagonal BC_2_P is computed from first-principles by deriving harmonic (2nd order) and anharmonic (3rd order) interatomic force interactions from first-principles and using them along with an exact solution of the Phonon Boltzmann Transport Equation (PBTE). We find an anisotropic high thermal conductivity (*k*) of 162 W m^−1^ K^−1^ and 52 W m^−1^ K^−1^ along directions perpendicular and parallel to *c*-axis (shown in Fig. 1a) respectively, at 0 GPa. Interestingly, optical phonons are found to contribute 30.1% (∼50 W m^−1^ K^−1^) and ∼15% (∼7.54 W m^−1^ K^−1^) at 300 K, to overall thermal conductivity along directions perpendicular and parallel to *c*-axis, respectively, due to their high group velocities. Finally, a high *k* value of 68 W m^−1^ K^−1^ at nanometer length scale of 100 nm (at 300 K) shows that BC_2_P will be a promising material for thermal management in nanoelectronics.

## Computational methods

First principles calculations were performed using QUANTUM ESPRESSO^24^ package. Thermal conductivity was computed by solving Phonon Boltzmann Transport Equation (PBTE) exactly using a variational method. The most important ingredients necessary to predict thermal conductivity, namely the 2nd order and 3rd order interatomic force constants (IFCs), were derived from density-functional perturbation theory (DFPT). These force constants are the second and third-order derivatives of energy with respect to atomic displacements. Computations were performed using norm-conserving pseudopotentials and exchange-correlation was computed in the local density approximation.^25^ The geometry of the hexagonal BC_2_P with 4 atoms unit cell, was optimized until forces on all atoms were less than 10^−6^ Ry per bohr. Plane-wave energy cutoff of 80 Ry and 12 × 12 × 8 Monkhorst–Pack^26^*k*-point mesh were used for electronic structure calculations. Optimized lattice constant (crystal structure of Fig. 1) of BC_2_P was obtained to be *a* = 5.2686 bohr with *c*/*a* = 1.686.

Elastic constants were computed using ‘thermo_pw’ package in QUANTUM-ESPRESSO;^24^ Voigt–Reuss–Hill approximation^27^ was used to calculate bulk modulus, shear modulus (*G*) and Young's Modulus (*E*). Lattice thermal conductivity is calculated by solving the Phonon Boltzmann Transport Equation (PBTE)^28–30^ exactly. Expression for thermal conductivity (*k*) obtained by solving PBTE in the single mode relaxation time (SMRT) approximation^31^ is given by,1
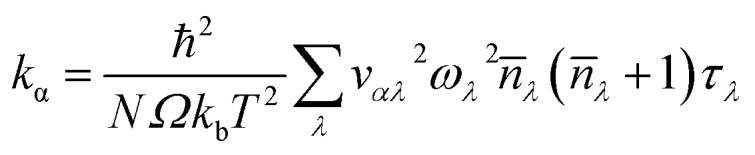
where, *α*, ℏ, *N*, *Ω*, *k*_b_, *T*, are the cartesian direction, Planck constant, size of the ***q*** mesh, unit cell volume, Boltzmann constant, and absolute temperature respectively. *λ* represents the vibrational mode (***q****j*) (***q*** is the wave vector and *j* represent phonon polarization). *ω*_*λ*_, *n̄*_*λ*_, and *v*_*αλ*_ (=∂*ω*_*λ*_/∂*q*) are the phonon frequency, equilibrium Bose–Einstein population and group velocity along cartesian direction *α*, respectively of a phonon mode *λ*. *ω*_*λ*_, *n̄*_*λ*_, and *c*_*αλ*_ are derived from the knowledge of phonon dispersion computed using 2nd order IFCs. *τ*_*λ*_ is the phonon lifetime and is computed using the following equation,2
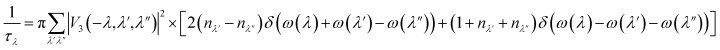
where, 
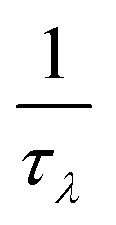
is the anharmonic scattering rate based on the lowest order three phonon interactions and *V*_3_(−λ, λ′, λ′′) are the three-phonon coupling matrix elements computed using both harmonic and anharmonic interatomic force constants. Harmonic force constants were calculated using 9 × 9 × 6 ***q***-grid. Anharmonic force constants were computed on a 3 × 3 × 2 ***q*** point grid using D3Q^28,30,32^ package within QUANTUM-ESPRESSO. Acoustic sum rules were imposed on both harmonic and anharmonic interatomic force constants. Phonon linewidth and lattice thermal conductivity were calculated using ‘thermal2’ code within QUANTUM ESPRESSO. For these calculations, a 21 × 21 × 14 ***q***-mesh was used and iterations in the exact solution of the PBTE were performed until Δ*k* between consecutive iterations diminished to below 1.0 × 10^−5^. Casimir scattering^33^ is imposed to include the effect of boundary scattering for computing length dependent thermal conductivity.

## Results and discussions

**Fig. 2:**
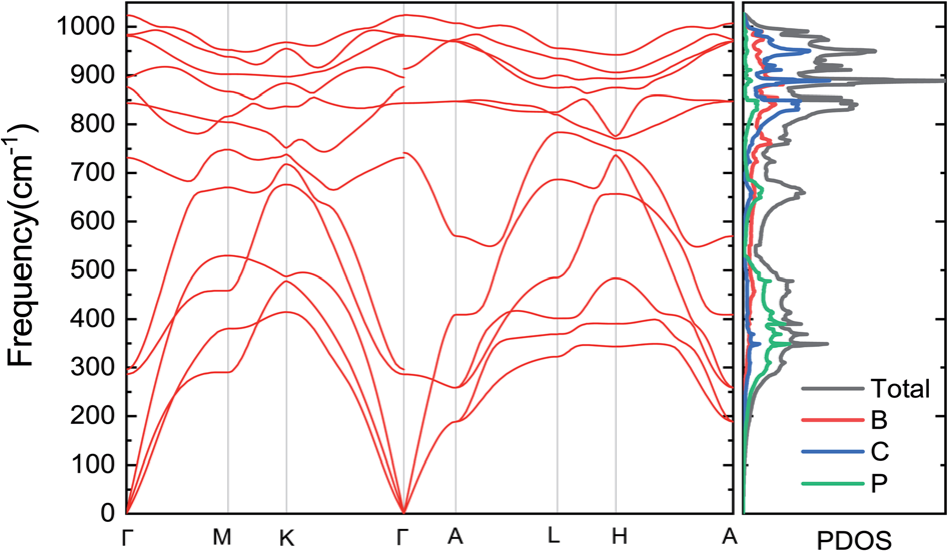
Phonon dispersion and phonon density of states for the h-BC_2_P along the high symmetry points. Red, blue and green in PDOS represent the vibrational frequencies of B, C and P atoms respectively.

Phonon dispersion and phonon density of states for hexagonal BC_2_P are shown in Fig. 2. Positive phonon frequencies indicate stability^34^ of computed h-BC_2_P crystal structure. Phonon modes at higher frequencies (above 750 cm^−1^) are mainly dominated by C and B atoms due to light mass and stiff C–C and B–C bonds, whereas P atoms dominate lower frequencies (less than 500 cm^−1^) due to heavy mass and moderate bond strengths of B–P and C–P. Elastic constants of hexagonal BC_2_P at 0 GPa are computed to be, C_11_ = 675 GPa, C_33_ = 680.6 GPa, C_44_ = 198 GPa, C_66_ = 305 GPa, C_12_ = 65.0 GPa and C_13_ = 30.8 GPa which satisfies the Born stability criteria^35^ of C_66_= (C_11_ − C_12_)/2, C_11_ > C_12_, C_33_(C_11_ + C_12_) > 2(C_13_),^2^ C_44_ > 0, C_66_ > 0. Young modulus (*E*), bulk modulus (*B*), shear modulus (*G*) and poisson ratio based on Voigt–Ruess–Hill approximation^27^ are 582.2 GPa, 253.6 GPa, 260.6 GPa and 0.117 respectively. These values are higher than silicon,^36^ germanium^36^ and silicon carbide.^37^

**Fig. 3:**
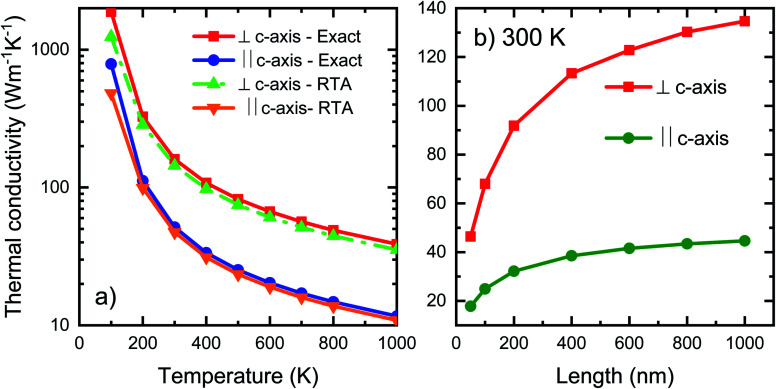
(a) Temperature dependence and (b) length dependence of thermal conductivity along and perpendicular to *c*-axis of h-BC_2_P.

**Fig. 4:**
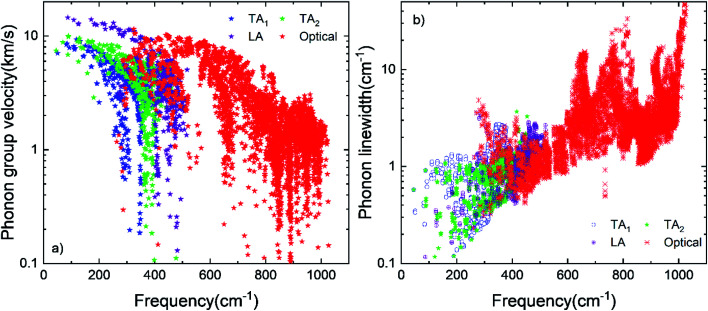
(a) Phonon group velocity and (b) phonon linewidth of TA, LA and optical phonon modes of h-BC_2_P at 300 K.

Computed thermal conductivity of the h-BC_2_P is reported in Fig. 3. Fig. 3a shows the temperature dependent thermal conductivity of h-BC_2_P along directions perpendicular and parallel to *c*-axis. At 300 K, computed thermal conductivity of 162 W m^−1^ K^−1^, perpendicular to *c*-axis, is almost 3 times higher than the value, parallel to *c*-axis, of 52 W m^−1^ K^−1^. This is due to the higher phonon frequencies of TA, LA and optical phonons modes, in a direction perpendicular to *c*-axis, relative to parallel to *c*-axis, as seen in the computed phonon dispersion. Thermal conductivity of h-BC_2_N is also higher than that of silicon.^38^ Perpendicular to *c*-axis, TA_1_, TA_2_ and LA phonon modes contribute 20.1%, 27.5% and 22.3% to overall thermal conductivity while along *c*-axis, the corresponding contributions are 23%, 30% and 32% to overall *k* at 300 K. Interestingly, at 300 K, optical phonon modes contribute 30.1% and 15% to overall thermal conductivity, along directions perpendicular and parallel to *c*-axis, respectively. This contribution is significantly higher than typical semiconductor materials such as silicon, where optical phonons contribute ∼5% to overall *k*. This is due to the high phonon group velocities of optical phonons (Fig. 4a) and optical phonon linewidths being comparable to that of acoustic phonons, in the frequency range of ∼300–550 cm^−1^ (Fig. 4b) in BC_2_P.

An advantage of BC_2_P is its relatively high thermal conductivity at nanometer length scales. Length dependence of thermal conductivity was calculated by introducing Casimir scattering 1/*τ*_boundary_ = |*v*|/*L*, where *v* is the phonon velocity and *L* is the system size. Length dependent thermal conductivity is shown in Fig. 3b. We observe that at a length scale of 100 nm, the predicted thermal conductivity of ∼68 W m^−1^ K^−1^ is significantly high. This can lead to potential avenues for use of BC_2_P in nanoscale thermal management applications. This high nanoscale thermal conductivity of BC_2_P is due to the relatively large *k* contribution of optical phonons, which typically have meanfreepaths in the nanometer regime.

## Conclusion

In this work, thermal conductivity of hexagonal BC_2_P (h-BC_2_P) is computed by solving phonon Boltzmann transport equation exactly coupled with force-constants derived from first principles calculations. We report an anisotropic thermal conductivity (*k*) of 162 W m^−1^ K^−1^ and 52 W m^−1^ K^−1^ along directions perpendicular and parallel to *c*-axis of BC_2_P respectively. This high thermal conductivity is due to the high frequencies and phonon group velocities arising from light mass of the constituent atoms (B, C, P) and stiff C–C, B–C and B–P bonds. Anisotropy in *k* is due to higher phonon frequencies and group velocities along direction perpendicular to *c*-axis relative to the parallel direction. Moreover, optical phonon modes are found to contribute significantly to *k* along directions both perpendicular to *c*-axis (30.1%) and parallel to *c*-axis (15%) at 300 K. Finally, a high room temperature thermal conductivity of 68 W m^−1^ K^−1^ at 100 nm length scale, makes BC_2_P attractive for thermal management in nanoelectronics.

## Conflicts of interest

There are no conflicts to declare.

## Supplementary Material

RA-010-D0RA08444A-s001
