# Supplementary material for: Thermal conductivity of hexagonal BC2P – a first-principles study
Source: RSC Adv. 2020 Nov 23;10(70):42628–32. doi: 10.1039/d0ra08444a (PMC9058011; doi:10.1039/d0ra08444a)
Supplement: RA-010-D0RA08444A-s001 [file RA-010-D0RA08444A-s001.pdf]

Electronic Supplementary Material (ESI) for RSC Advances.
This journal is © The Royal Society of Chemistry 2020

Supplementary Information

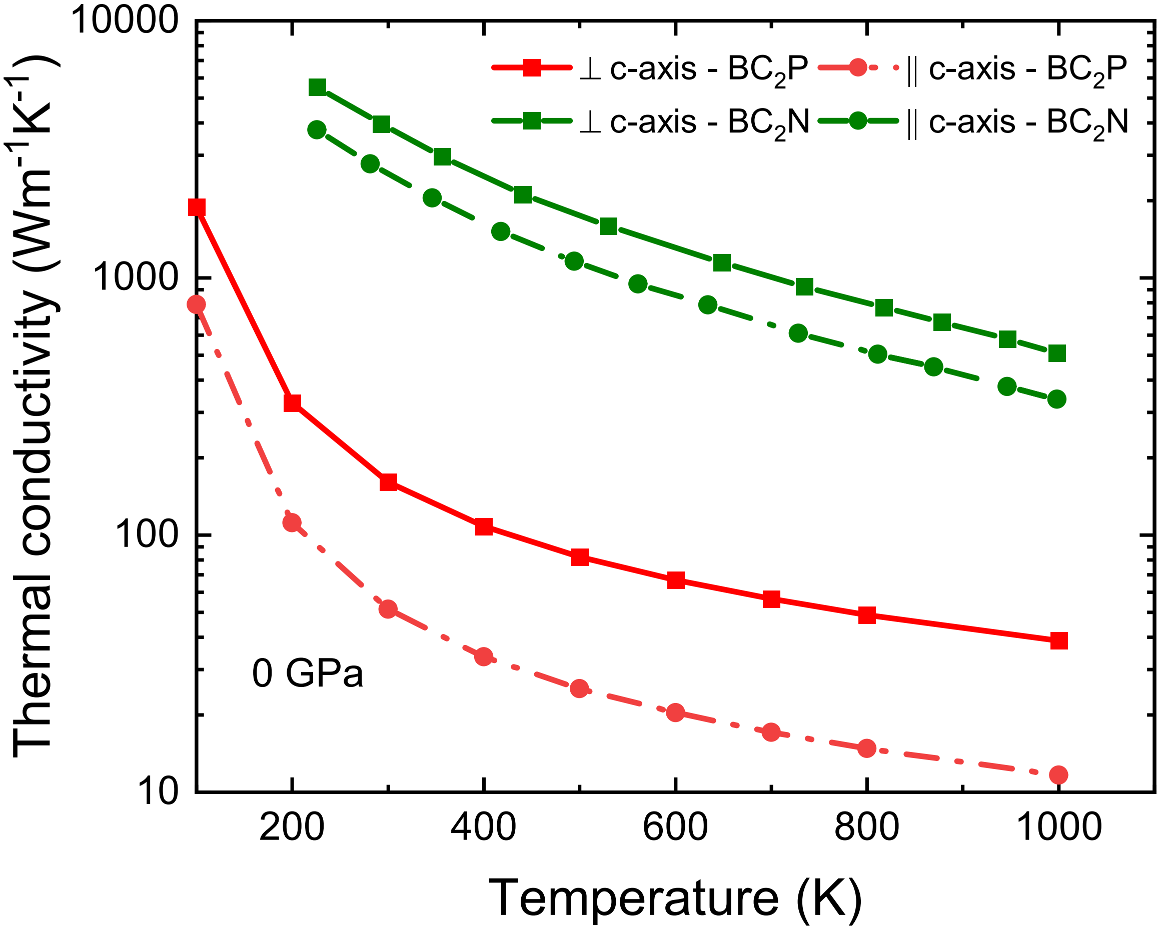

Figure S1: Thermal conductivity of hexagonal BC_2P and BC_2N along in-plane and out-of-plane directions at 0 GPA
